# Supplementary figures and images for: Biology, ecology, and biogeography of eremic praying mantis Blepharopsis mendica (Insecta: Mantodea)
Source: PeerJ. 2024 Jan 29;12:e16814. doi: 10.7717/peerj.16814 (PMC10832664; doi:10.7717/peerj.16814)

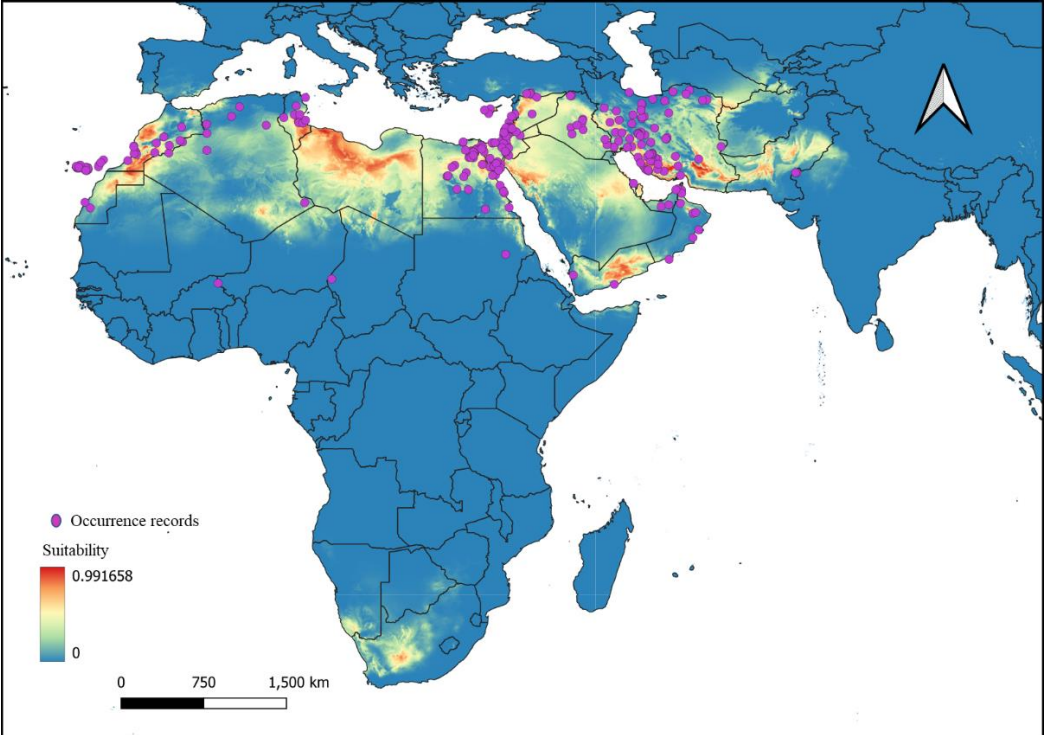

Supplement: Supplemental Information 1 [file peerj-12-16814-s001.pdf]

Supplementary material Figure S2: Phylogenetic analyses of COI sequences.

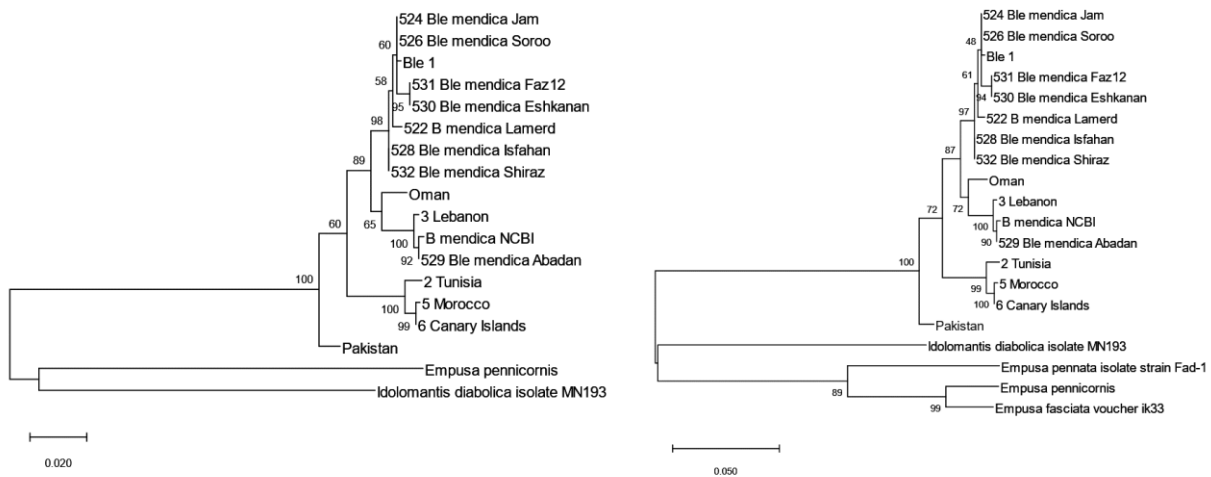

Supplement: Supplemental Information 2 [file peerj-12-16814-s002.pdf]
