# Supplementary material for: Biology, ecology, and biogeography of eremic praying mantis Blepharopsis mendica (Insecta: Mantodea)
Source: PeerJ. 2024 Jan 29;12:e16814. doi: 10.7717/peerj.16814 (PMC10832664; doi:10.7717/peerj.16814)
Supplement: Supplemental Information 3 [file peerj-12-16814-s003.docx]

**Supplementary material table S1:** Information regarding the samples used in this study

| **Species** | **Latitude** | **Longitude** | **voucher** | **Accession No.** | **Haplotype No.** |
| --- | --- | --- | --- | --- | --- |
| *Blepharopsis mendica* | 27.3417 | 53.0892 | ZMPC522 | OR588779 | H_01 |
| *Blepharopsis mendica* | 27.8399 | 52.052483 | ZMPC524 | OR588780 | H_01 |
| *Blepharopsis mendica* | 29.5694 | 51.947 | ZMPC526 | OR588781 | H_02 |
| *Blepharopsis mendica* | 32.8343 | 51.749 | ZMPC528 | OR588782 | H_05 |
| *Blepharopsis mendica* | 30.5694 | 48.9002 | ZMPC529 | OR588783 | H_07 |
| *Blepharopsis mendica* | 27.2204 | 53.6006 | ZMPC530 | OR588784 | H_03 |
| *Blepharopsis mendica* | 27.7996 | 52.1264 | ZMPC531 | OR588785 | H_05 |
| *Blepharopsis mendica* | 29.4731 | 52.1679 | ZMPC532 | OR588786 | H_04 |
| *Blepharopsis mendica* | 29.1766 | 53.3804 | ESPCBle1 | OR588787 | H_01 |
| *Blepharopsis mendica* | 35.6683 | 10.11 | RB#2 | OR588788 | H_12 |
| *Blepharopsis mendica* | 34.3978 | 36.3868 | RB#3 | OR588789 | H_08 |
| *Blepharopsis mendica* | 32.4293 | -4.4707 | RB#5 | OR588790 | H_11 |
| *Blepharopsis mendica* | 28.7233 | -13.8758 | RB#6 | OR588791 | H_10 |
| *Blepharopsis mendica* | 19.5839 | 54.8827 | VG#1 | OR588792 | H_06 |
| *Blepharopsis mendica* | 24.75 | 69.7667 | MAN-00032 | BOLD:AAW0516 | H_09 |
