# Supplementary material for: Biology, ecology, and biogeography of eremic praying mantis Blepharopsis mendica (Insecta: Mantodea)
Source: PeerJ. 2024 Jan 29;12:e16814. doi: 10.7717/peerj.16814 (PMC10832664; doi:10.7717/peerj.16814)
Supplement: Supplemental Information 4 — Sample sizes (n), standard deviations (SD), standard errors (SE), confidence intervals (CI), t-values, degrees of freedom (DF). [file peerj-12-16814-s004.docx]

**Supplementary material table S2:** comparative summary of adult longevity between males and females of *Blepharopsis mendica*. Sample sizes (n), standard deviations (SD), standard errors (SE), confidence intervals (CI), t-values, degrees of freedom (DF).

|  | **Males** | **Females** |
| --- | --- | --- |
| n | 17 | 17 |
| Mean | 46.63 | 118.41 |
| SD | 4.7 | 6.4 |
| SE | 1.14 | 1.55 |
| CI | 43.16-50.10 | 114.18-122.65 |
| t-value | -29.33 | 29.33 |
| DF | 32 |  |
| P-value | <0.001 |  |
