# Supplementary material for: Biology, ecology, and biogeography of eremic praying mantis Blepharopsis mendica (Insecta: Mantodea)
Source: PeerJ. 2024 Jan 29;12:e16814. doi: 10.7717/peerj.16814 (PMC10832664; doi:10.7717/peerj.16814)
Supplement: Supplemental Information 5 [file peerj-12-16814-s005.docx]

**Supplementary material table S3:** Comparison of Nymph to Death Duration Between Males and Females of *Blepharopsis mendica.*

|  | **Sample Size** | **Sample Mean** | **Sample Variance** | **t-Statistic** | **Degrees of Freedom (df)** | **p-value** |
| --- | --- | --- | --- | --- | --- | --- |
| Nymph to Death Males | 17 | 132.06 | 78.48 | 1.12 | 16 | 0.279 |
| Nymph to Death Females | 17 | 216.18 | 133.14 |  |  |  |
