# Supplementary material for: Biology, ecology, and biogeography of eremic praying mantis Blepharopsis mendica (Insecta: Mantodea)
Source: PeerJ. 2024 Jan 29;12:e16814. doi: 10.7717/peerj.16814 (PMC10832664; doi:10.7717/peerj.16814)
Supplement: Supplemental Information 6 [file peerj-12-16814-s006.docx]

**Supplementary material table S4:** List of the new record of *B. mendica* from this study

| **Province** | **District name** | **latitude** | **longitude** |
| --- | --- | --- | --- |
| Bushehr Province | Ab Pakhsh | 29.367532 | 51.047097 |
| Bushehr Province | Bandar Siraf | 27.681073 | 52.327239 |
| Bushehr Province | Dorahak | 27.942029 | 51.952182 |
| Bushehr Province | Jam | 27.813106 | 52.290029 |
| Bushehr Province | Khormoj | 28.671837 | 51.357423 |
| Bushehr Province | Nakhl-e ghanem | 27.805803 | 52.11942 |
| Chaharmahal and Bakhtiari Province | Chah Gorg | 32.211342 | 51.107986 |
| Chaharmahal and Bakhtiari Province | Farokhshahr | 32.237017 | 51.023287 |
| Chaharmahal and Bakhtiari Province | Naqneh | 31.944519 | 51.314474 |
| Chaharmahal and Bakhtiari Province | Tang-e-sayad National Park | 32.15559 | 51.116381 |
| Fars Province | Atashkadeh | 28.904304 | 52.545012 |
| Fars Province | Barmshour Sofla | 29.452825 | 52.698433 |
| Fars Province | Bidak | 29.147232 | 53.421446 |
| Fars Province | Dowlat Abad | 29.271593 | 53.161963 |
| Fars Province | Gerash | 27.658293 | 54.207292 |
| Fars Province | Heshmatiye | 31.159294 | 52.600774 |
| Fars Province | Islam Abad | 29.423554 | 52.179007 |
| Fars Province | Jahrom | 28.520629 | 53.498024 |
| Fars Province | Khonj | 27.901771 | 53.406165 |
| Fars Province | Marvdasht | 29.845526 | 52.766038 |
| Fars Province | Meymand | 28.877213 | 52.759676 |
| Fars Province | Sadra | 29.746691 | 52.638175 |
| Fars Province | Zarqan | 29.734662 | 52.758517 |
| Golestan Province | Turkmen Sahra | 37.28899 | 55.268614 |
| Hamadan Province | Karimabad | 34.930683 | 48.411512 |
| Hormozgan Province | Hajiabad | 28.290841 | 55.887283 |
| Hormozgan Province | Qalatoo | 27.179419 | 56.189564 |
| Isfahan Province | Aran o Bidgol | 34.048852 | 51.520433 |
| Isfahan Province | Aran o Bidgol | 34.267344 | 51.806968 |
| Isfahan Province | Golpayegan | 33.423942 | 50.308039 |
| Isfahan Province | Naein County | 32.874192 | 53.047456 |
| Isfahan Province | Shahreza | 32.059731 | 51.88823 |
| Isfahan Province | Zarinshahr | 32.405494 | 51.393589 |
| Kerman Province | Bam | 29.149359 | 58.364106 |
| Kerman Province | Sirjan | 29.46797 | 55.569068 |
| Khuzestan Province | Ahvaz | 31.32079 | 48.709631 |
| Khuzestan Province | Behbahan | 30.594014 | 50.286257 |
| Khuzestan Province | Ghale Took | 32.384079 | 48.340446 |
| Khuzestan Province | Khorramshahr | 30.576544 | 48.165474 |
| Khuzestan Province | Lali | 32.313096 | 49.099305 |
| Khuzestan Province | Mal Agha | 31.595085 | 50.028003 |
| Khuzestan Province | Omidiyeh | 30.554135 | 49.754033 |
| Kohgiluyeh and Boyer-Ahmad Province | Sisakht | 30.882791 | 51.456191 |
| Lorestan Province | Romeshkan | 33.333111 | 47.369274 |
| Mazandaran Province | Mangol | 36.383002 | 52.840108 |
| Qom Province | Salafchegan | 34.471404 | 50.442017 |
| Razavi Khorasan Province | Neyshabur | 36.223139 | 58.850756 |
| Razavi Khorasan Province | Torghabeh | 36.302052 | 59.363831 |
| Semnan Province | Damghan | 36.155469 | 54.284144 |
| Yazd Province | Fahraj | 31.760263 | 54.569438 |
| Yazd Province | Mehriz | 31.550348 | 54.463279 |
| Yazd Province | Mehriz | 31.583606 | 54.392321 |
