# Supplementary material for: Biology, ecology, and biogeography of eremic praying mantis Blepharopsis mendica (Insecta: Mantodea)
Source: PeerJ. 2024 Jan 29;12:e16814. doi: 10.7717/peerj.16814 (PMC10832664; doi:10.7717/peerj.16814)
Supplement: Supplemental Information 7 — The semi-axes length are represented by a, b, and c, and the axes coordinates are represented by the vectors vec_1 and vec_2. The covariance matrix shows the variances and covariances among the principal components (pc_1, pc_2, pc_3). [file peerj-12-16814-s007.docx]

**Supplementary material table S5:** Metadata obtained from *Ellipsoid niche model*, the results obtained by the MVE1 method at level 99, including the centroid coordinates, covariance matrix, volume, semi-axes length, and axes coordinates. The semi-axes length are represented by a, b, and c, and the axes coordinates are represented by the vectors vec_1 and vec_2. The covariance matrix shows the variances and covariances among the principal components (pc_1, pc_2, pc_3). The volume is given in arbitrary units.

|  | **pc_1** | **pc_2** | **pc_3** |
| --- | --- | --- | --- |
| Centroid | 1.344 | -2.046 | 0.604 |
| Covariance | 0.134 | 0.134 | 0.161 |
|  | 0.134 | 0.521 | 0.350 |
|  | 0.161 | 0.350 | 0.422 |
| Volume | 13.462 |  |  |
| Semi-axes | a | b | c |
| Length | 0.818 | 1.230 | 3.165 |
| Axes | pc_1 | pc_2 | pc_3 |
| Coordinates |  |  |  |
| a | 2.036 | -1.928 | 0.184 |
|  | 0.651 | -2.163 | 1.025 |
| b | 1.916 | -2.887 | 1.312 |
|  | 0.772 | -1.204 | -0.103 |
| c | 0.499 | -4.325 | -1.424 |
|  | 2.188 | 0.233 | 2.633 |
